# Fast Prediction of Deterioration and Death Risk in Patients With Acute Exacerbation of Chronic Obstructive Pulmonary Disease Using Vital Signs and Admission History: Retrospective Cohort Study

**DOI:** 10.2196/13085

**Published:** 2019-10-21

**Authors:** Mi Zhou, Chuan Chen, Junfeng Peng, Ching-Hsing Luo, Ding Yun Feng, Hailing Yang, Xiaohua Xie, Yuqi Zhou

**Affiliations:** 1 Surgical Intensive Care Unit The Third Affiliated Hospital of Sun Yat-sen University Guangzhou China; 2 School of Data and Computer Science Sun Yat-sen University Guangzhou China; 3 Department of Respiratory and Critical Care Medicine The Third Affiliated Hospital of Sun Yat-sen University Guangzhou China

**Keywords:** chronic obstructive pulmonary disease, clinical decision support systems, health risk assessment

## Abstract

**Background:**

Chronic obstructive pulmonary disease (COPD) has 2 courses with different options for medical treatment: the acute exacerbation phase and the stable phase. Stable patients can use the Global Initiative for Chronic Obstructive Lung Disease (GOLD) to guide treatment strategies. However, GOLD could not classify and guide the treatment of acute exacerbation as acute exacerbation of COPD (AECOPD) is a complex process.

**Objective:**

This paper aimed to propose a fast severity assessment and risk prediction approach in order to strengthen monitoring and medical interventions in advance.

**Methods:**

The proposed method uses a classification and regression tree (CART) and had been validated using the AECOPD inpatient’s medical history and first measured vital signs at admission that can be collected within minutes. We identified 552 inpatients with AECOPD from February 2011 to June 2018 retrospectively and used the classifier to predict the outcome and prognosis of this hospitalization.

**Results:**

The overall accuracy of the proposed CART classifier was 76.2% (83/109 participants) with 95% CI 0.67-0.84. The precision, recall, and F-measure for the mild AECOPD were 76% (50/65 participants), 82% (50/61 participants), and 0.79, respectively, and those with severe AECOPD were 75% (33/44 participants), 68% (33/48 participants), and 0.72, respectively.

**Conclusions:**

This fast prediction CART classifier for early exacerbation detection could trigger the initiation of timely treatment, thereby potentially reducing exacerbation severity and recovery time and improving the patients’ health.

## Introduction

### Background

Chronic obstructive pulmonary disease (COPD) is characterized by incomplete reversible airflow obstruction. Patients with COPD may experience exacerbations of the disease, which are associated with significant morbidity and mortality as well as reduced quality of life. COPD is a serious long-term condition that progressively restricts airflow from the lungs and imposes a significant burden on patient’s daily lives [1]. Currently, it is the fourth leading cause of death in the world but is projected to be the third by 2030 [2-4]. As one of the most common and frequently occurring diseases, COPD has 2 different courses: the acute exacerbation phase and the stable phase. An acute exacerbation of COPD (AECOPD) has been described as an acute worsening of respiratory symptoms associated with a variable degree of physiological deterioration [5]. Sudden deterioration because of any cause requires critical medical care and may require hospitalization. Previous studies have shown that early intervention on these COPD patients decreases morbidity of acute exacerbation and mortality [6].

Since 2001, according to the Global Initiative for Chronic Obstructive Lung Disease (GOLD) guideline, patients with stable COPD have been classified as mild, moderate, severe, and extremely severe depending on lung function. The 2011 GOLD guideline has been revised to divide patients with COPD into grades A, B, C, and D. This classification method has been improved several times and is still in use today, which is based on lung function, frequency of acute exacerbations, symptom scores, and risk factors [7]. However, AECOPD patients are highly heterogeneous. According to the differences in basic conditions, causes, and complications, the acute exacerbations of different patients in the same grade may be different, and even the 2 consecutive acute exacerbations of the same patient may be very different. Patients with mild acute exacerbation may be discharged after several days of treatment; however, patients with severe acute exacerbation may require longer hospital stay, higher costs, ICU admission, and even mechanical ventilation. In the worst case, a small number of patients may eventually die without remission. Therefore, it is important to assess the severity of acute exacerbations in patients with COPD, which can determine what treatments are needed to improve prognosis and reduce mortality [8,9]. However, there is currently no consensus on the assessment of the severity of acute exacerbations.

There are some attempts to predict the course of disease using machine learning in general and deep learning models in particular. Most of the studies analyzed the correlation between clinical treatment and prognosis. Amalakuhan et al [10] took advantage of random forest (RF) algorithm to research which patients were at high risk for multiple COPD exacerbations and hospital readmission within a single year. The study included 60 indicators in 106 patients, such as medical history, general conditions, and medication, and the prediction accuracy is 0.72. However, because patients have many influencing factors outside the hospital, such as weather changes, environmental pollution, treatment compliance, and pathogen epidemics, this may affect the accuracy of prediction. Yang et al [11] used 3 methods to predict the risk of 30-day readmission of patients. The study used a public database with a total of 323,813 patients and 100 features, and COPD patients were a subgroup among them. The precision rate was 0.257, and the recall rate was 0.786. Zheng et al [12] proposed a hesitant fuzzy linguistic complex proportional assessment method to solve the decision-making problems under hesitant fuzzy linguistic environment. The study assessed the severity of COPD patients by outpatient doctors’ description of patient symptoms and risk factors, but it was difficult to verify the accuracy of the evaluation because of lack of follow-up and prognostic data. Swaminatha et al [13] collected vital signs, symptoms, and comorbidities data of patients with COPD. The study used physician opinion in a statistically and clinically comprehensive set of patient cases to train a supervised prediction algorithm. After 2400 training sessions, the gradient-enhanced RF algorithm was 88% identical to the physician’s judgment in 101 validated cases. However, the study also lacked follow-up outcome data, making it difficult to verify whether the subjective judgment of the physician met the objective prognosis.

### Objectives

Although scholars have worked on predicting the severity of AECOPD with various machine learning algorithms, none of the abovementioned studies examined the fast severity assessment approach, which only requires the patient’s vital signs and admission history data that can be collected within minutes after admission. In this study, we propose a fast severity assessment and risk prediction approach by exploring the usefulness of the classification and regression tree (CART) for fast predicting the severity of AECOPD once the patient is admitted to the hospital. CART as a decision tree algorithm was introduced by Leo Breiman in 1985, which is successfully used for classification or regression predictive modeling problems [14]. The proposed fast assessment system can help the doctors to obtain the severity assessment of the patients quickly within minutes after admission. The fast prediction CART classifier is a promising research tool for the identification of at-risk populations with COPD. Therefore, it is necessary to establish a rapid classification method to predict the outcome and prognosis of patients with AECOPD.

## Methods

### Data Acquisition

The data of AECOPD patients were obtained from the Department of Respiratory and Critical Care Medicine of the Third Affiliated Hospital, Sun Yat-sen University (TAHSYU). TAHSYU is a comprehensive third-grade class-A hospital directly managed by the National Health Commission of the People’s Republic of China. We searched for medical records of all inpatients from 2011 to 2018, screening out patients with a major diagnosis of AECOPD by using International Classification of Diseases, Tenth Revision, Clinical Modification (ICD-10-CM) code for AECOPD (J44.100, J44.101). Patients needed to have a pulmonary function test record with forced expiratory volume in 1 second/forced vital capacity <0.7, and the main complaint in this hospitalization included a description of increased cough or shortness of breath. We excluded the patients who were discharged without medical advice or had missing variables. Finally, 552 hospitalized AECOPD patients were included. For statistical purposes, the triage was labeled as mild group and severe group according to the situation of the patient during hospitalization. Here, mild group means the patient was stable and no intensive care was required, eventually got better, and was discharged. Severe group means the patient had a notable deterioration, needed intensive care, was dead, dying, incurable, and automatically discharged. The distribution of AECOPD patients with mild and severe symptoms is shown in Table 1. The research was performed under the guidance of the TAHSYU Institutional Review Board, protocol #2019-02-334-01.

**Table 1:** Distribution of mild and severe groups in patients with acute exacerbation of chronic obstructive pulmonary disease.

Characteristic		Mild group (low risk)	Severe group (high risk)
Number of cases, n (%)		308 (55.8)	244 (44.2)
**Outcome, n (%)**			
	Dead	0 (0.0)	24 (9.8)
	Deteriorating discharge	0 (0.0)	50 (20.4)
**Sex, n (%)**			
	Male	240 (77.9)	201 (82.4)
	Female	68 (22.1)	43 (17.6)
Smoking history, n (%)		215 (69.8)	167 (68.4)
Age (year), mean (SD)		75.06 (7.94)	76.66 (9.49)
Number of hospitalizations, mean (SD)		4.21 (4.07)	5.97 (6.87)
Days in hospital, mean (SD)		8.01 (2.64)	73.96 (195.21)
Costs (RMB Yuan), mean (SD)		9428 (2921)	68,860 (98,109)

### Data Analysis

The GOLD guideline is only for the classification of patients in stable phase, and there is no consistent classification for patients with acute exacerbations. Some scholars have proposed a 2-axis and 4-group classification by considering the pathobiological and clinical heterogeneity of AECOPD [15], but it takes a long time to get results and also requires more clinical validation. Thus, we propose a fast assessment indicator system to make it more reasonable and practical by the advice of the clinician.

One of the important missions is the variable selection in the process of fast assessing the severity of the COPD. In this paper, the process of predictor selection is shown as follows:

- Step 1: Find some predictors from the perspective of system engineering;
- Step 2: Make sure the predictors can be collected quickly after the patient is admitted to the hospital;
- Step 3: Verify the reasonability of the above predictors from the clinical experience of professional pulmonary physicians;
- Step 4: Predictors with too many missing values (more than 10% over 552 rows of records) are discarded directly to avoid inaccurate predictions;
- Step 5: Laboratory testing data are not included because most laboratory testing results are not available within 10 min, such as blood gas analysis, sputum culture, and so on;
- Step 6: Text-based features like common chief complaints that need to be processed by natural language processing are left for subsequent processing and are not included now.

From the abovementioned steps, we can establish a fast assessment system that only includes these 7 variables at the beginning of admission. In particular, this indicator can be obtained within minutes after admission.

1. Respiratory rate (RR): RR is one of the most important predictors of the COPD, and excessive breathing rate is the main factor causing the patients feeling anxious with the loss of physical ability [16]. Normal respiration rate is between 12 and 18 breaths per minute. Typical COPD patients describe excessive breathing rate as a sense of shortness of breath, wheezing, or needing great effort to breathe.
2. Systolic blood pressure (SBP) and diastolic blood pressure (DBP): Blood pressure is usually expressed in terms of SBP over DBP and is measured in millimeters of mercury (mmHg), reflects the stability of the blood circulation. Blood pressure in patients with severe COPD may be affected by hypoxemia or cardiac insufficiency.
3. Pulse rate (PR): Pulse is also one of the important indicators for doctors to diagnose COPD. The PR changes obviously when the patient is in critical condition. Therefore, measuring PR is an indispensable examination item for patients.
4. Number of hospitalizations (NOH): NOH is defined as the total number of hospitalizations of patients at TAHSYU. NOH is proportional to the severity of the disease. Generally, the greater the number of admissions, the more severe the COPD patient will be.
5. Temperature (TEMP): Body temperature is an important indicator of body metabolism, which is dynamically balanced within a certain range. COPD patients often develop fever because of inflammation. Especially, the measurement of the patients’ TEMP is relatively simple and rapid.
6. Smoking: Define a patient who has smoked for 6 consecutive months as having a history of smoking. Smoking is one of the most common risk factors of COPD and will worsen the severity of COPD.

### Mode Selection

CART is a nonparametric statistical procedure containing classification procedure and regression procedure. It is formed by using a set of if-then-else logical conditions to assign an unknown vector of feature values (or predictors) to a predefined class or category. CART methodology has been increasingly applied to health sciences and clinical research and has been applied to a much lesser extent in COPD condition monitoring. Algorithms for constructing a CART usually work top down, by choosing a variable at each step that best splits the set of items [17]. Gini impurity, information gain, and variance reduction are often applied to each candidate subset, and the resulting values are combined (eg, averaged) to provide a measure of the quality of the split [18].

The results calculated by CART techniques are straightforward to interpret. Compared with the black box model, such as neural network algorithm, CART is a highly interpretive model. Compared with the white box model, such as linear regression, CART does not need data to satisfy the linear priori hypothesis. In addition, CART analysis has the statistical advantage of being a nonparametric technique that does not invoke assumptions about the functional form of the data. Furthermore, CART can process multiclass problem easily. Finally, CART is good at processing categorical and missing features easily and nonlinear test efficiently.

### Classification Using a Classification and Regression Tree

At this stage, 7 predictors collected from 552 COPD patients’ records included NOH, smoking history, RR per minute, TEMP, PR, SBP, and DBP. From the available dataset, each of the *N* observations is denoted by the 2-tuple, (*x, y*), where *x* ∈{*x*_1_*,x*_2_*,...,x*_7_} is the vector containing all the 7 features. *y* ∈{1*,*2} represents the categories of low risk and high risk.

In the process of mode training, we use 80% of the observations for model training and the remaining 20% for mode validation. A cross-validated grid-search approach is employed to tune the hyperparameters of the CART. To avoid overtraining the CART, we first estimate the optimal depth of the CART. The tree depth is defined as the maximum number of branches (a branch joins 2 nodes) on the path from any leaf node to the root node. The tree construction algorithm is described as follows: (1) search the best predictor as the root node of the tree according to gini index. The node is then split using the best predictor to create 2 leaf nodes; for multivariate classification, all variables are evaluated by gini values to find the variable with the minimum gini values as the root node of the CART. Gini index is usually selected as the measurement for the classification problem to reduce a chosen global measure of impurity for the tree; the messier the category overall, the bigger the gini index; (2) if the node is no longer separable, then the node is stored as a leaf node; a completely pure node contains only instances from 1 class; (3) the splitting process is repeated (binary splitting) until all leaf nodes reside no greater than the predefined depth from the root node for all existing leaf nodes [19,20]; (4) create left and right subtrees recursively.

In the process of the model testing, we measure the classification performance of CART model by precision, recall, and F-Measure. We check the prediction performance of the model on the training set and the test set to choose the best model by avoiding overfitting and underfitting. We implemented CART classifier on the development platform of R 3.5.1. R is available as free software in source code form. It was originally developed at Bell Laboratories by John Chambers and colleagues, which provides a wide variety of statistical and graphical techniques and is highly extensible.

## Results

### The Accuracy of Classifier

Precision, recall, and F-measure are the measures widely used in the field of information retrieval and statistical classification to evaluate the quality of results. Precision is defined as the ratio of the correct number predicted by the model to the actual correct number. Recall is defined as the ratio of the actual correct number to the correct number predicted by the model. F-measure is the weighted average of precision and recall. The larger the parameters are, the better the prediction performance will be. In particular, 1 is the ideal state.

**Figure 1:**
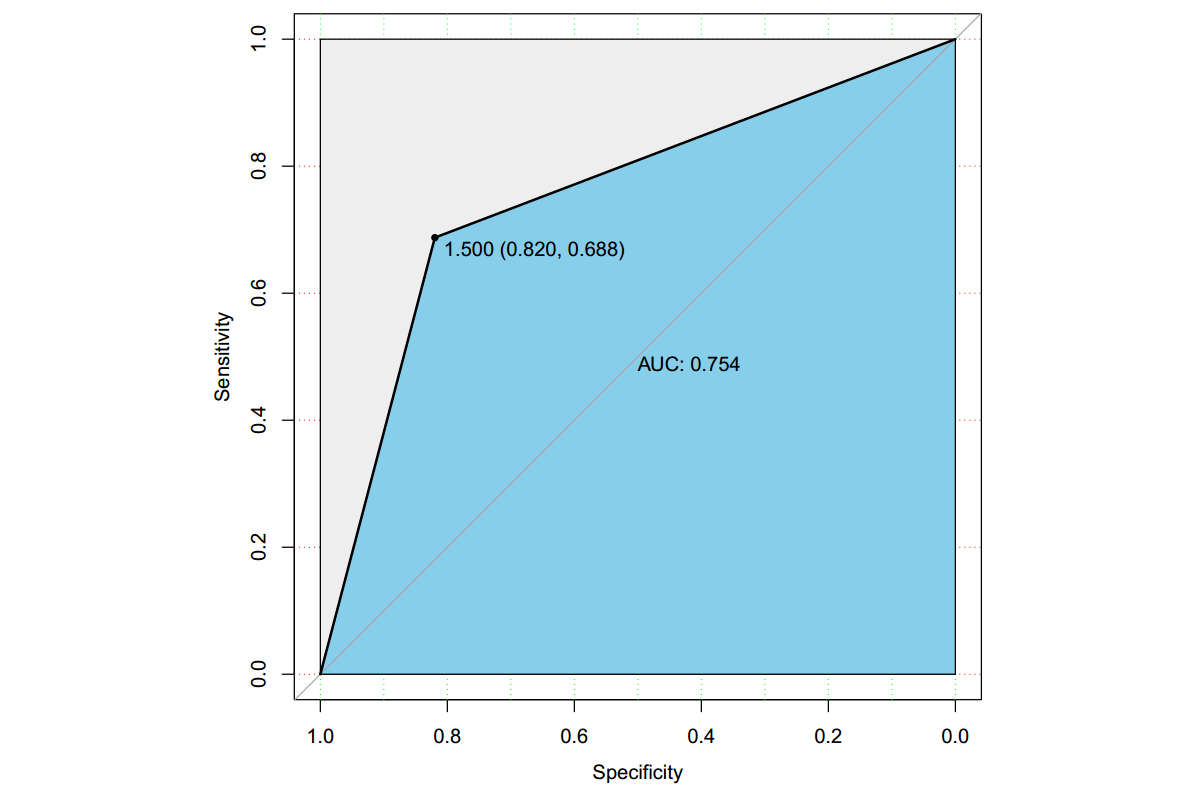
Receiver operating characteristic curve in the classification and regression tree classifier. AUC: area under the curve.

The overall accuracy on the test dataset of the proposed CART classifier was 76.2%, with 95% CI 0.67-0.84. The evaluation of the fast prediction CART classifier is shown in Table 2. The receiver operating characteristic curve of the CART classifier is shown in Figure 1. The optimal tipping point is 1.50 (0.82, 0.69). The area under the curve is 0.75.

Currently, the proposed CART classifier can achieve the same performance on a similar test dataset. However, to improve the generalization of the model, it is necessary to provide a wide variety of training samples to gain more comprehensive knowledge. By working with external data sources, we can provide a more comprehensive set of training for the model, allowing the model to gain more comprehensive knowledge and continuously improve predictive performance. In addition, we use the K-fold cross verification method to estimate the depth of the CART tree.

**Table 2:** Evaluation of fast prediction classification and regression tree classifier on test dataset. The overall accuracy was 76.2%.

Group	Precision, %	Recall, %	F-measure
Mild group (low risk)	76	82	0.79
Severe group (high risk)	75	68	0.72

### The Importance of Variables

To understand the contribution of each predictor to the CART model, we computed the variable importance in the tree model. Table 3 shows the variable importance of the fast prediction CART classifier. The predictor is more important if the value on the x-axis is bigger. We can find that RR per minute, SBP, PR, DBP, NOH, TEMP, and smoking history (Smoking) were important predictors. The RR per minute reflects the severity of dyspnea and may be a good indicator of prognosis [21]. Other vital signs also reflect the overall condition of the patient. For example, elevated TEMP may mean more serious infections, increased heart rate may represent severe dyspnea, or heart failure. Hypertension is one of the most common comorbidities in COPD patients; all of the above are related to the prognosis of the patient [22,23].

**Figure 2:**
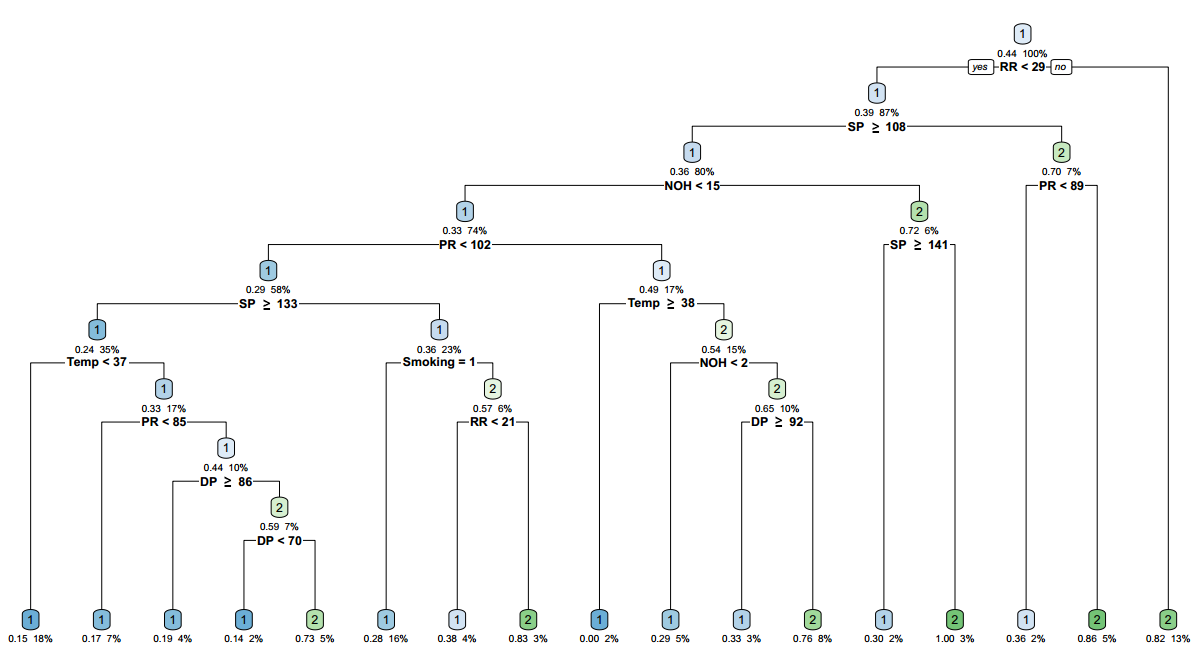
The classification and regression tree constructed from one run. DP: diastolic pressure; NOH: number of hospitalizations; PR: pulse rate; RR: respiratory rate; SP: systolic pressure; Temp: temperature.

This CART model for early detection could trigger the initiation of timely treatment, thereby potentially reducing exacerbation severity and recovery time and improving the patients’ health. Figure 2 is an illustration of a CART constructed from one run. A leaf node in the tree, 1 represents a low-risk health prediction and 2 represents high-risk one. Each decision node represents the corresponding feature used and the decision threshold. At each decision node, the left branch is chosen if the feature is less than the threshold value. RR is the root node of the tree. The closer the root node is, the more important the feature is. We can see from Figure 2 that if the RR of a patient with COPD is greater than 29 breaths per minute, the patient is a high-risk patient; otherwise, it can be judged according to other features, and so on.

**Table 3:** The relative importance of each variable to the prediction, with respiratory rate as 100%.

Variables	Relative importance (%)
Respiratory rate	100.0
Systolic blood pressure	85.8
Pulse rate	68.6
Diastolic blood pressure	66.1
Number of hospitalizations	59.2
Temperature	49.0
Smoking	22.8

## Discussion

### Clinical Significance

In this study, we investigated approaches to fast predict the severity and risk of patients with AECOPD within minutes after admission. Fast predicting can serve as a useful tool for physicians to assess the risk of deterioration, thereby strengthening monitoring and medical interventions in advance. CART classifier proposed in this paper predicts 76.2% of instances correctly.

The clinical presentation and disease progression of AECOPD patients are significantly heterogeneous, that means the severity of patients is quite different. Severe patients may need to be admitted to the ICU with systemic glucocorticoids, broad-spectrum antibiotics, or even mechanical ventilation [24,25]. Therefore, it is important to judge the severity and prognosis of patients with AECOPD early. However, the GOLD guideline is only for the treatment of patients in stable phase, and there is no consistent classification for patients with acute exacerbations. Some scholars have proposed a 2-axis and 4-group classification by considering the pathobiological and clinical heterogeneity of AECOPD [15], but it also requires more clinical validation.

Machine learning provides a powerful tool for the classification and prediction of COPD patients, but the pathogenesis of COPD is not completely clear, the course of the disease is extremely complex. Therefore, the data need to be properly selected and analyzed to obtain more accurate and credible conclusions. The patients in this study had an exact outcome and were hospitalized for standardized treatment, effectively reducing the impact of factors outside the hospital. The selection of features and the time span of observation are also very important, and there are many factors that influence the prognosis of patients with AECOPD. Multiple studies have attempted to predict risk factors that affect mortality and readmission rates in AECOPD patients, such as acute physiology and chronic health evaluation scores, C-reactive protein, blood carbon dioxide partial pressure, and blood urea nitrogen [26,27]. Obviously, incorporating more features and increasing observation time is beneficial to improve the accuracy of the forecast, but it also increases the cost and complexity of the assessment. If a large number of examinations and several days of time are needed for prediction, the clinical significance will become very poor.

The 7 indicators we selected are simple, fast, noninvasive, and objective. Measurements only require watches, sphygmomanometers, and thermometers. In clinical work, usually the nurses measure vital signs, ask for general information, and register after admission, which takes 7 to 10 min. If we only specifically acquire the 7 indicators and use an electronic sphygmomanometer and an infrared thermometer, the time can be shortened to less than 3 min. Doctors, nurses, and even trainees can quickly grasp the assessment method. This is very helpful in assessing the severity and risk of patients before the senior physician arrives or in scheduling the intensive care unit resources faster. Although this study included hospitalized patients, it can be applied to outpatients or even patients for self-assessment because features can be easily and quickly obtained.

### Limitations

In addition to the 7 indicators of this study, there are some other indicators that can be quickly obtained and may be helpful in predicting prognosis. Cough, dyspnea, and increased sputum are criteria for judging acute exacerbations, and the severity of these symptoms correlates with prognosis. However, most of the hospitalized patients in this study have these symptoms to varying degrees, and it is difficult to quantify the changes and severity of these symptoms in the medical records. Some studies use a sound monitoring system to continuously record a patient’s cough and perform an automated analysis to assess the severity of cough [28]. However, it is still difficult to guide clinical practice in the short term.

Complications, such as acute heart failure and diabetes, may significantly increase mortality in patients with AECOPD [29]. However, for newly hospitalized patients, multiple tests and several days may be required to diagnose the comorbidities, which limits the rapid judgment of prognosis. For patients with repeated hospitalizations and chronic comorbidities with a clear history, this may be more meaningful. We will use text mining methods to improve data and further study the impact of chronic comorbidities on the prognosis of patients with AECOPD.

There are also some shortcomings in this study. Due to the single-center study, the number of cases is small. The research also lacks oxygen saturation data, which is a simple, noninvasive indicator of oxygenation in patients. This is because in the past few years, not all patients, especially those with mild symptoms, have routinely measured blood oxygen saturation. If oxygen saturation is included, the amount of data will be significantly reduced, while causing bias. Now, with the popularity of portable finger oximeters, the vast majority of patients measure blood oxygen saturation on admission, which can be used in subsequent studies.

In summary, this study shows that the use of machine learning methods to analyze the vital signs and other indicators of newly hospitalized patients may help clinicians to judge the severity of patients more quickly, so as to carry out early medical intervention for patients with severe AECOPD. In spite of this, the results are still valid when some of the variables are not included, as this study is not a causal analysis, but an exploratory data analysis. The proposed model is generic enough to cope with similar medical scenarios, provided that these data can be obtained as long as COPD patients are hospitalized.

### Conclusions

In this study, we developed a fast severity assessment and risk prediction approach, which only requires the patient’s vital signs and admission history data that can be collected within minutes, and showed that it can rapidly predict the severity of COPD patients. The overall accuracy of the proposed CART classifier is 76.2% with 95% CI 0.67-0.84. It is concluded that CART classifier can be used as a forecasting tool for COPD inpatients. As CART is a nonlinear system, it is found that its performance is better than previous classifiers or regression techniques. Further work can be done on similar lines by adding predictors, or optimizing the classifier parameters, or using other fusion learning algorithms, such as RF [30].

## Abbreviations

AECOPD: acute exacerbation of chronic obstructive pulmonary disease
CART: classification and regression tree
COPD: chronic obstructive pulmonary disease
DBP: diastolic blood pressure
GOLD: Global Initiative for Chronic Obstructive Lung Disease
NOH: number of hospitalizations
PR: pulse rate
RF: random forest
RR: respiratory rate
SBP: systolic blood pressure
TAHSYU: Third Affiliated Hospital, Sun Yat-sen University
TEMP: temperature
